# Supplementary material for: PARP1 Regulates Circular RNA Biogenesis though Control of Transcriptional Dynamics
Source: Cells. 2023 Apr 14;12(8):1160. doi: 10.3390/cells12081160 (PMC10136798; doi:10.3390/cells12081160)
Supplement: Supplementary file 1 [file cells-12-01160-s001.zip › Eleazer et al, 2023-Supplemental Figures-dg.pdf]

Supplemental Figures

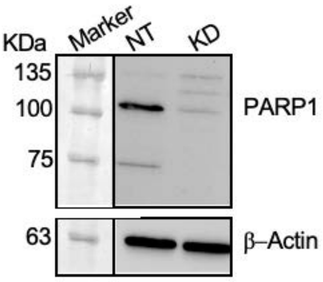

Figure S1. Validation of PARP1-KD. Depletion of PARP1 expression in S2 cells by siRNA. ~~**(A)**~~ PARP-1 protein expression measured by Western blot. β-actin used as loading control.

A

B

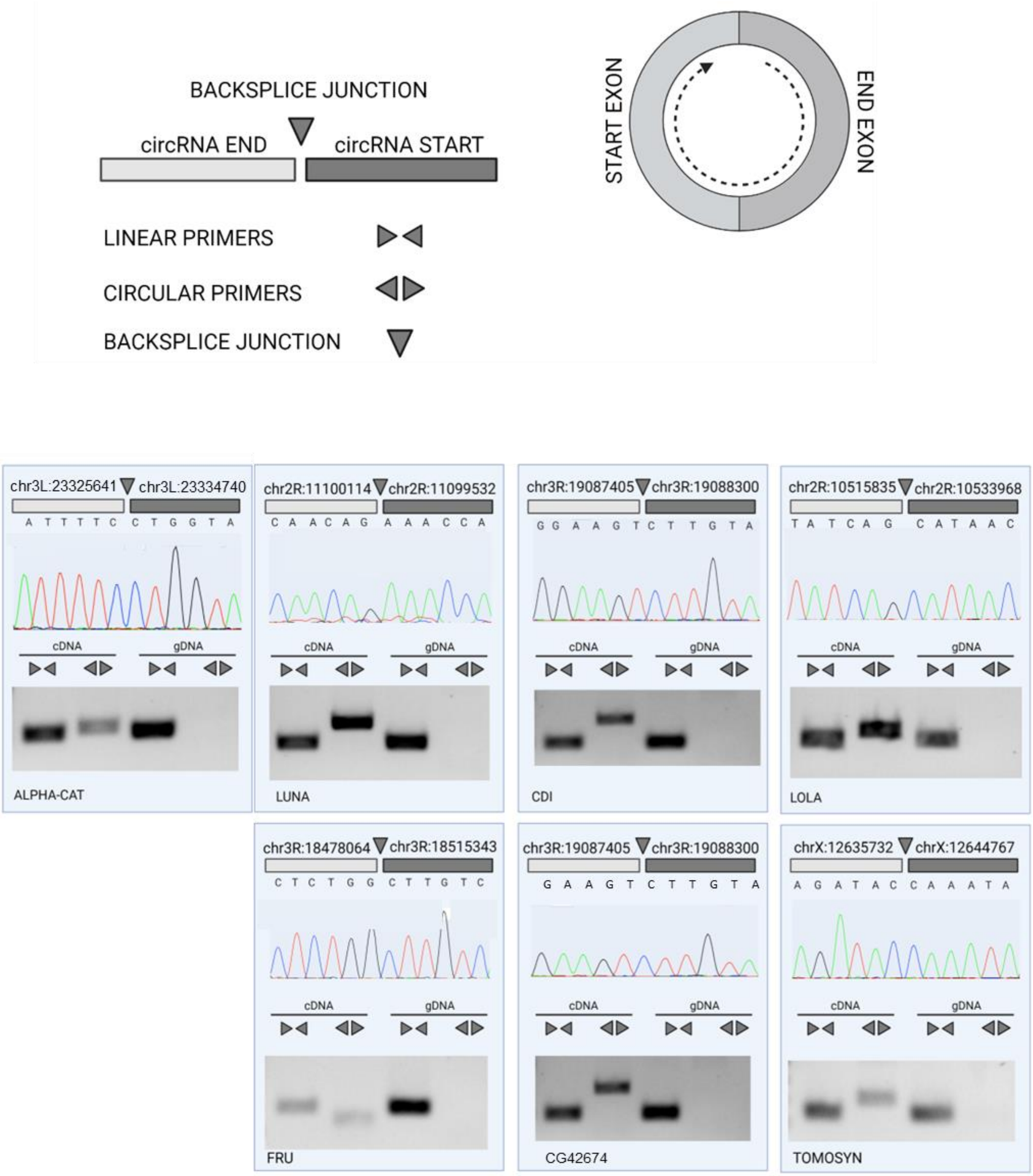

Figure S2. Validation of selected circRNAs using PCR. Primers were designed to amplify the backsplice junction (demarked above) and a linear region of each circRNA. Convergent primers will amplify both circular transcripts and linear gDNA while divergent primers will only amplify the circular transcripts. **B**. PCRs were performed with these primer sets on cDNA generated from total RNA as well as genomic DNA. Bands from DNA amplified with diverging primers on cDNA were cut, purified, cloned and sequenced to confirm the presence of the backsplice junction (chromatograms are shown above the agarose gel images).

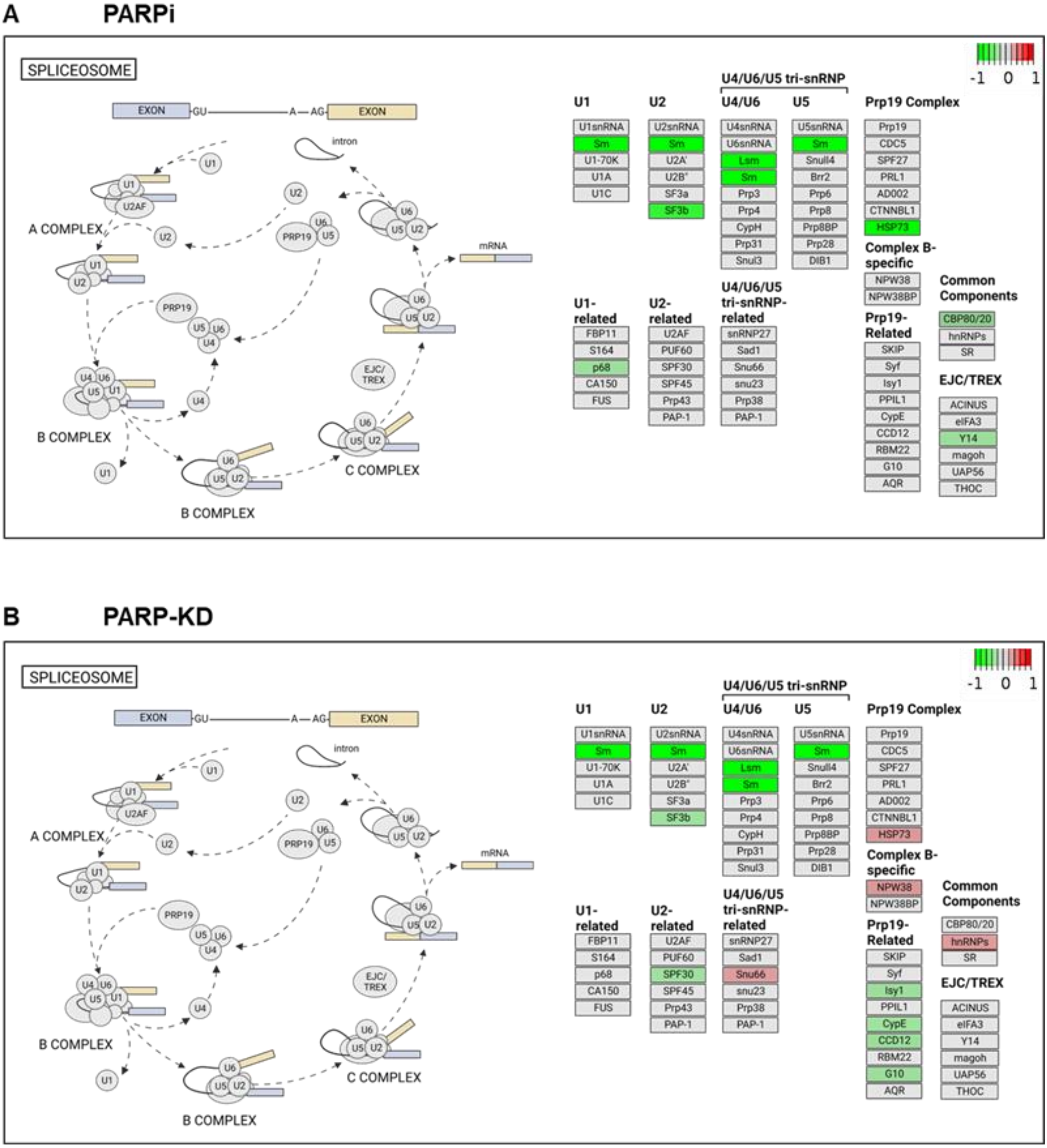

Figure S3. Loss of PARP1 and PARylation alters the expression of core spliceosomal components. Cartoon depicting the KEGG spliceosome pathway map of spliceosome. Highlighted are the significant DEGs (q<0.05) from RNA-seq analysis (Pathview Luo et al. 2017) of the spliceosomal components in PARPi (A) and PARP-KD(B). The spliceosomal components downregulated are shown in green while upregulated are shown in red.

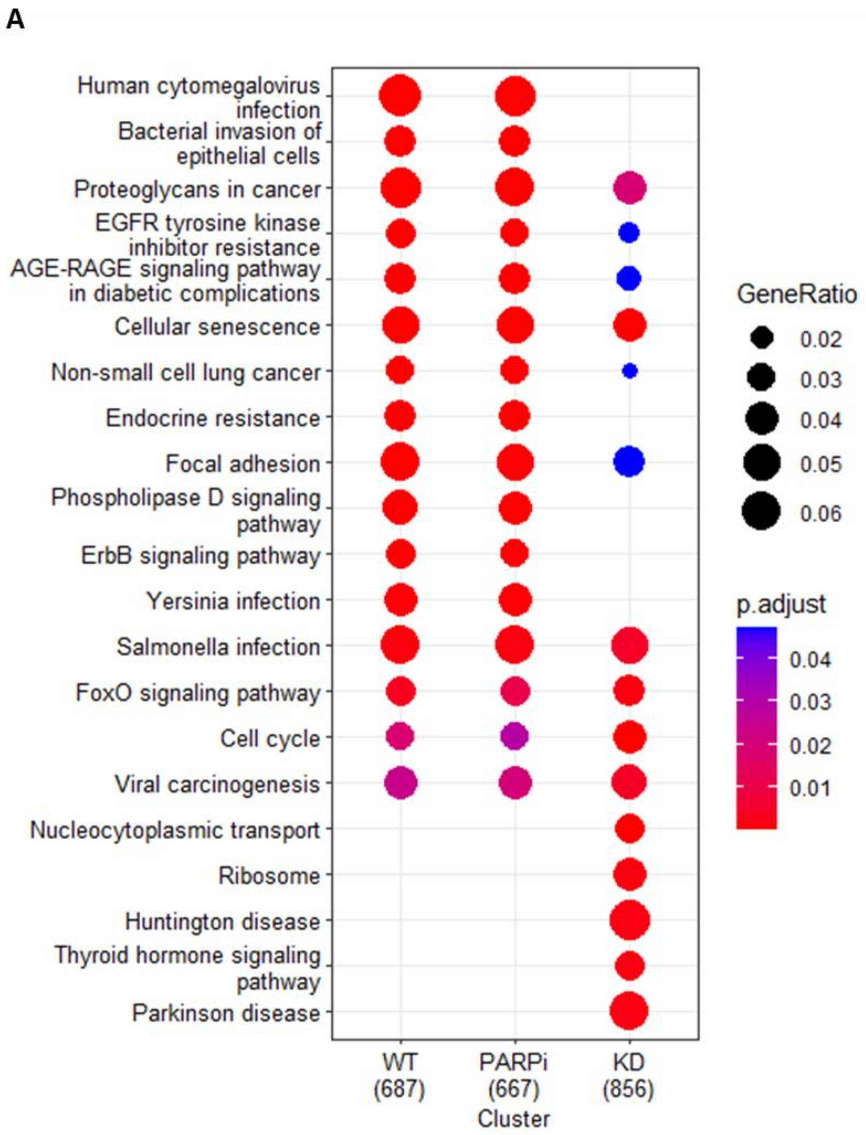

Figure S4. KEGG pathways predicted to be regulated by circRNA-miRNA-mRNA regulatory network. Dot plot comparison of differential KEGG pathways regulated by circRNA-microRNA predicted interactions for circRNAs unique for WT (column 1) PARPi (column 2) and PARP1 KD (column 3). The most frequent miRNA targets in each experiment circRNA group were identified from miRTarBase (Release 9.0) (Huang, Lin et al., 2022) relative to human miRNA seeds. The target functional annotation was completed using clusterProfiler.

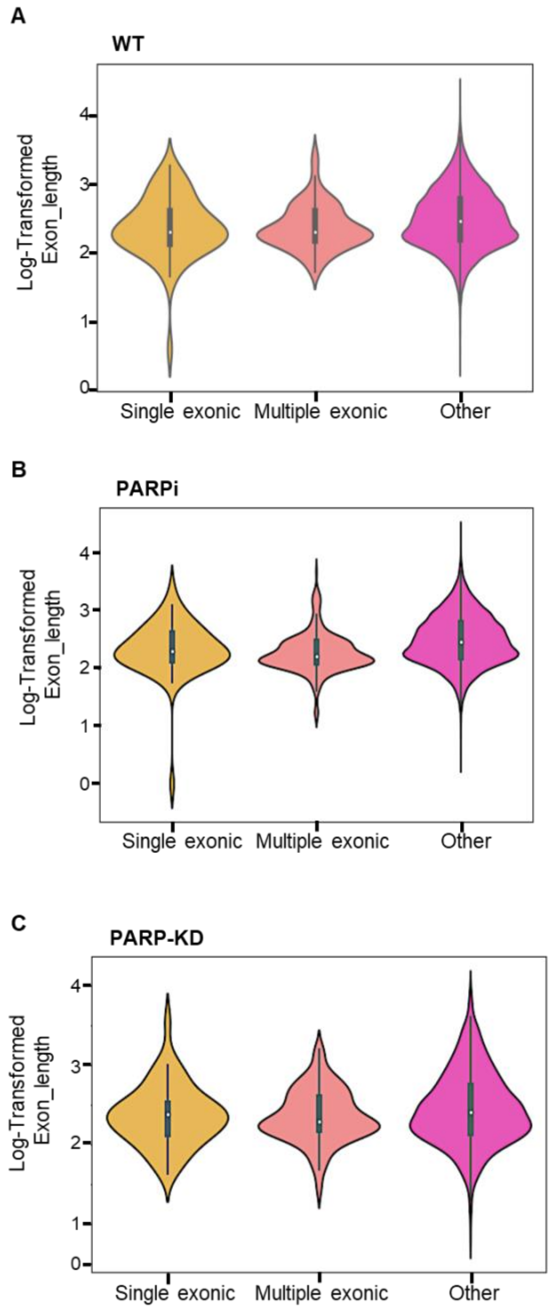

Figure S5. Exonic characteristics of circRNA host genes. Violin plots showing the distribution of exon lengths in WT (A), PARPi (B) and PARP-KD (C) circRNA. Exons from circRNA spanning a single exon (yellow) and exons from circRNA spanning multiple exons (salmon?) are compared to other Drosophila exons (pink).

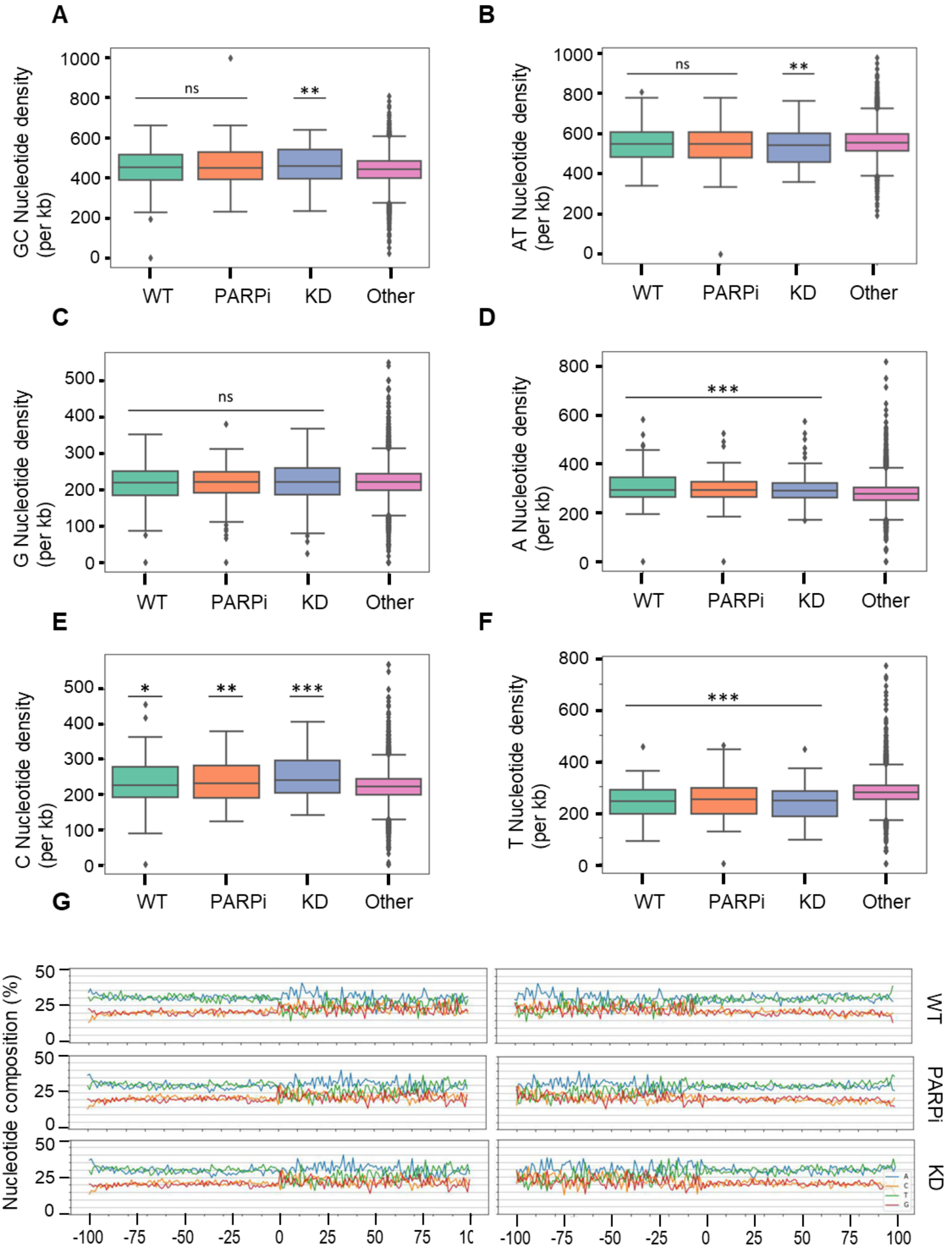

Figure S6. Atypical nucleotide composition of host genes. (A-F) circRNA host genes in WT (green) PARPi (orange) and KD (blue) are differentially enriched for A and C nucleotide compared to other Drosophila genes (pink). Shown are the nucleotide density for GC content (A) AT content (B) G content (C) A content (D) C content (E) and T content (F). Wilcoxon rank sum (P). ns P>0.05 *P<0.05 **P<0.01 ***P<0.001. (G) Metagene analysis showing the distribution of all four nucleotides from 1000bp of the upstream intron through the first 100bp of the circRNA acceptor exon (left) and the last 100 bp of the circRNA donor exon through 1000bp downstream into the intron (right).

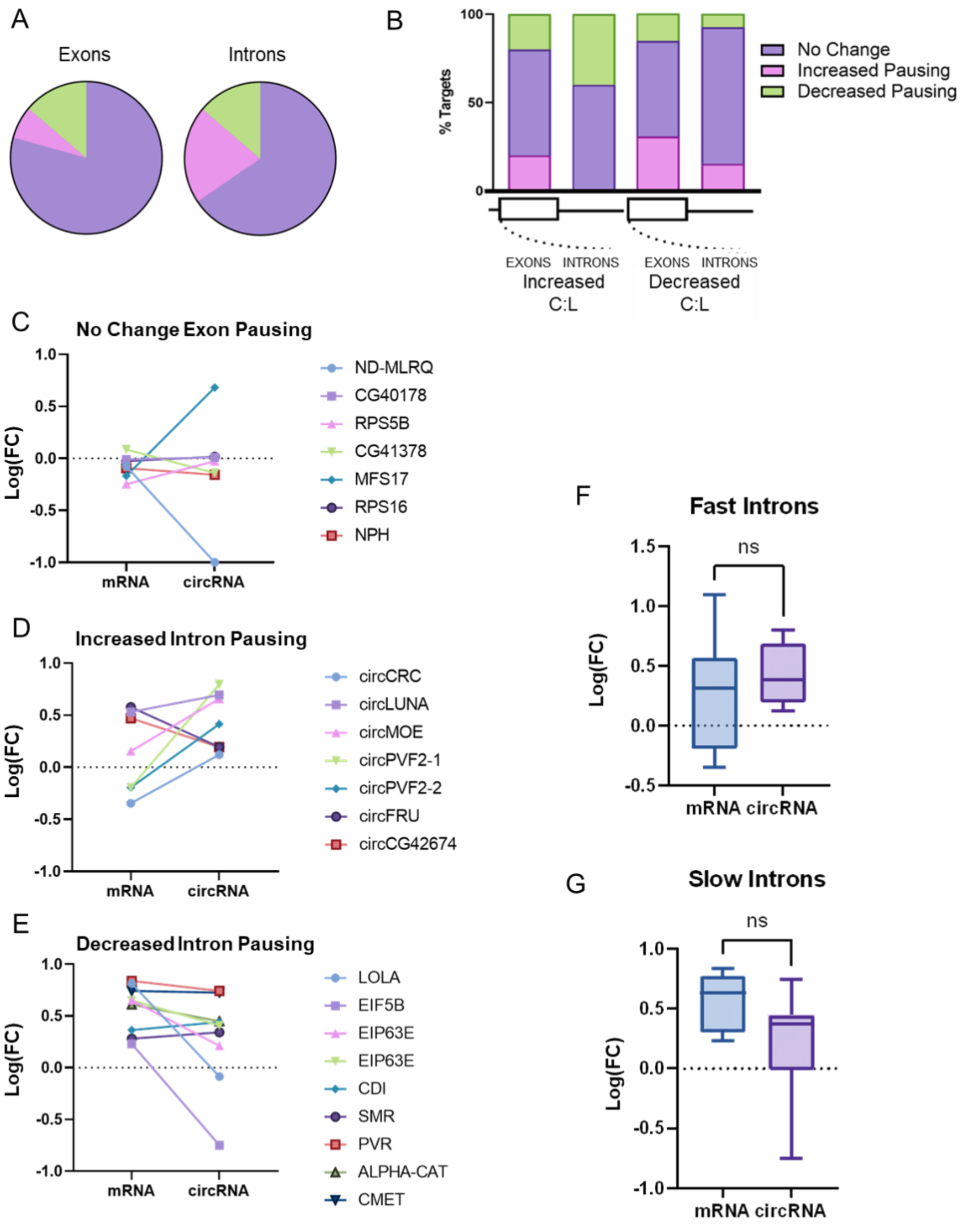

Figure S7. Pausing profiles of circRNA gene regions(A) Distribution of circRNA spanning exons (left pie chart) or introns (right pie chart) having increased pausing (pink), decreased pausing (green) or no change in pausing (purple) upon PARP1-KD. (B) Stacked bar graph showing the change in pausing within exons or introns spanning circRNA showing increased circular to linear (C;L) ratio compared to those host genes with decreased C:L ratio. (C-E) Paired log fold change (LogFC) of the expression of the mRNA and circRNA for each host gene with no change in RNAPII pausing within exons (C) increased pausing within introns (D) or decreased pausing within introns (E). LogFC of mRNA and circRNA for host genes with decreased pausing within introns (F) and increased pausing within introns (G).
